# Epigenetic modification of *ESP,* encoding a putative long noncoding RNA, affects panicle architecture in rice

**DOI:** 10.1186/s12284-019-0282-1

**Published:** 2019-04-03

**Authors:** Xin Luan, Shuchun Liu, Shanwen Ke, Hang Dai, Xin-Ming Xie, Tzung-Fu Hsieh, Xiang-Qian Zhang

**Affiliations:** 1 Guangdong Engineering Research Center of Grassland Science, College of Forestry and Landscape Architecture, South China Agricultural University, Guangzhou, 510642 China; 2 Plants for Human Health Institute, North Carolina State University, North Carolina Research Campus, Kannapolis, NC 28081 USA; 3 Department of Plant and Microbial Biology, North Carolina State University, Raleigh, NC 27695 USA

**Keywords:** Rice, lncRNA, Epigenetic, Panicle, Pseudogene

## Abstract

Epigenetic variants broaden phenotypic diversity in eukaryotes. Epialleles may also provide a new genetic source for crop breeding, but very few epialleles related to agricultural traits have been identified in rice. Here, we identified *Epi-sp*, a gain-of-function epiallele of the rice *ESP* (*Epigenetic Short Panicle*, *Os01g0356951*), which encodes a putative long noncoding RNA. The *Epi-sp* plants show a dense and short panicle phenotype, an agronomically important phenotypes that is inherited in a semidominant manner. We did not find any nucleotide sequence variation in *ESP*. Instead, we found hypomethylation in the transcriptional termination region (TTR) of *ESP* gene, which caused ectopic expression of *ESP* in *Epi-sp* plants. Bisulfite analysis revealed that the methylation status of 26 CGs and 13 CHGs within a continuous 313-bp region is essential for the regulation of *ESP* expression. Thus, our work identified a unique rice epiallele and demonstrated that epigenetic modification of *ESP* is associated with the regulation of panicle architecture in rice.

**Electronic supplementary material:**

The online version of this article (10.1186/s12284-019-0282-1) contains supplementary material, which is available to authorized users.

## Findings

In higher plants, epigenetic variation provides a mechanism for phenotypic variation in the absence of DNA mutations. Although a number of epigenetic mutants were identified in multiple plant species, including *Arabidopsis* (Jacobsen and Meyerowitz 1997; Soppe et al. 2000), *Linaria vulgaris* (Cubas et al. 1999), *Solanum lycopersicum* (Manning et al. 2006) and *Cucumis melo* (Martin et al. 2009), examples of epigenetic diversity in rice remain limited. The first epigenetic mutants identified in rice is the *Epi-d1*, that shows a metastable dwarf phenotype, often producing both dwarf and normal tillers on the same plant (Miura et al. 2009). Metastable phenotype of *epi-d1* is due to metastable expression of *DWARF1* (*D1*) gene, which is associated with DNA methylation in the *D1* promoter region. Other epigenetic mutants of rice such as *Epi-df* (Zhang et al. 2012), *Epi-RAV6* (Zhang et al. 2015), also show DNA hypermethylation or hypomethylation in the promoter of the corresponding genes, resulting in the suppression or induction of their transcription.

Previous studies have shown that epigenetic modifications can affect various aspects of plant growth and development including plant height, seed size, floral development and disease resistance in rice (Miura et al. 2009; Zhang et al. 2012; Zhang et al. 2015; Deng et al. 2017). Panicle architecture, an important agronomic trait of rice, is mainly determined by the number and length of the primary and secondary branches. Although a number of key genes controlling panicle architecture such as *DEP2* (Li et al. 2010) and *SP1* (Li et al. 2009) have been cloned and characterized in recent years, whether epigenetic modification is involved in the regulation of panicle architecture remain unclear. Here, we identified a natural epiallele of rice *ESP* and found that plants carrying this epiallele show reduced panicle size, as well as hypomethylation in the *ESP* transcriptional termination region (TTR) and ectopic expression of *ESP*. Our work revealed that epigenetic modification of *ESP* plays an important role in the regulation of panicle architecture in rice.

**Fig. 1:**
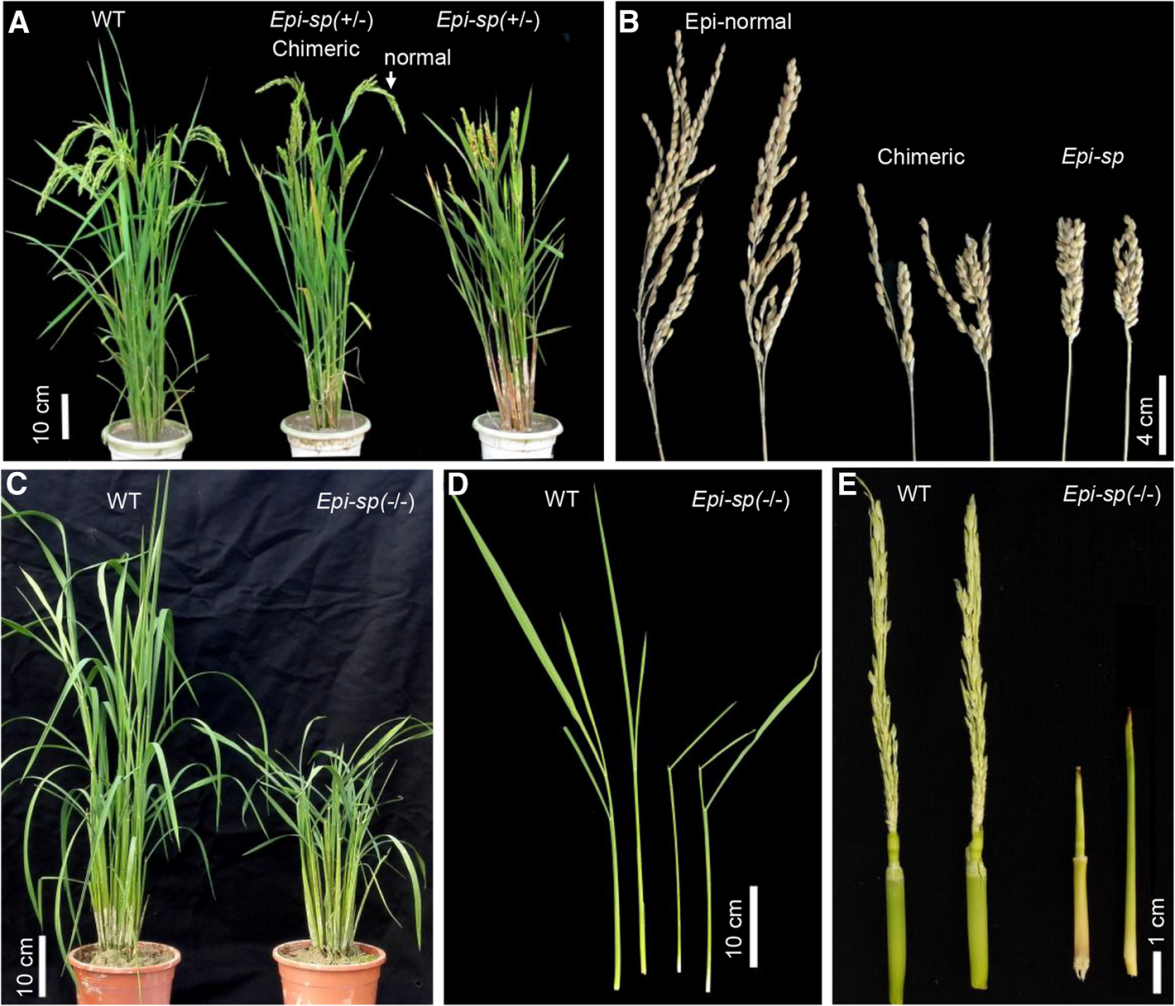
Characterization of a semidominant rice mutant with short panicle. **a** Gross morphology of *Epi-sp* mutant. **b** *Epi-sp* shows varying phenotypes in panicle. **c-e**, the homozygous *Epi-sp* mutant is defect in shoot apical meristem

A spontaneously occurring rice mutant with a dense and shortened panicle was isolated from the *japonica* rice ‘Zhonghua11’ and was named *Epi*-*sp* (*epigenetic short panicle*) based on our subsequent characterization (see below). To determine the inheritance of *Epi*-*sp*, we examined the phenotypes of 210 progeny of self-pollinated *Epi*-*sp* plants. Three distinct phenotypes were observed. Among them, 58 were normal looking (wild type) plants, 103 displayed shortened panicle, and 49 exhibited dwarf phenotypes (Fig. 1). The ratio of wild type to short panicle to dwarf phenotypes was 1:1.78:0.84 (for 1:2:1 ratio, χ^2^ = 0.85, *P* > 0.05). In addition, 35 F_1_ plants derived from the cross between *Epi-sp* and Zhonghua11 showed a near 1:1 ratio of wild-type vs short panicle progeny (Data not shown). These results demonstrated that the *Epi*-*sp* mutant is a heterozygous allele controlled by a single, semi-dominant locus. Thus, the short panicle progeny is referred to as the heterozygotes *Epi*-*sp* (*+/*−), and the dwarf progeny as the homozygotes *Epi*-*sp* (*−*/*−*).

The heterozygous *Epi*-*sp* (*+/*−) mutant showed a significant reduction in plant height (97.4 ± 3.4 cm), compared to the wild-type plants (106.7 ± 3.1 cm), resulting from significantly shortened panicles and branches in the mutant culms (Fig. 1B and Additional file 1: Fig. S1). But there were no significant differences in the number of primary and secondary branches and grains morphology between wild type and mutant plants (Additional file 1: Figure S1). The overall appearance of the mutant panicle is more compact compared to the wild type. By contrast, the normal tillers have normal features (Fig. 1a). Interestingly, the heterozygous *Epi*-*sp* (*+/*−) mutant was often chimeric, producing both dwarf (*Epi-sp*) and normal tillers (*Epi-normal*) on the same plant (Fig. 1a). In addition, we found chimeric features also in the panicles (Fig. 1b). This strongly suggests that the *Epi-sp* mutant phenotypes are not genetically fixed and can vary epigenetically in the developing plant. For the homozygous mutant, it exhibited a dwarf phenotype (24.8 ± 2.8 cm) with a defect in shoot apical meristem (SAM) and could not set seeds in paddy field (Fig. 1c-e).

**Fig. 2:**
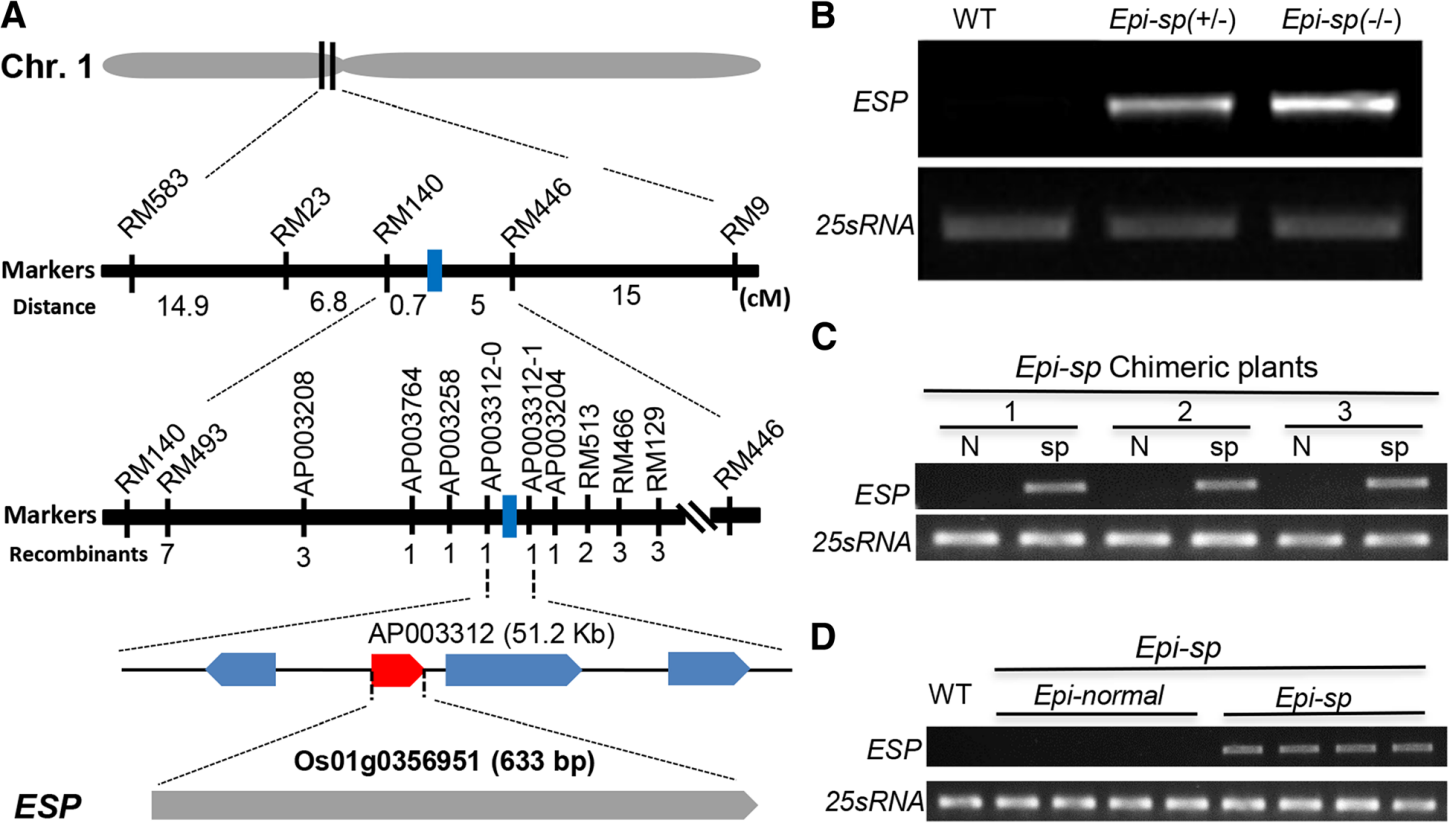
Molecular cloning and expression analysis of *ESP*. **a** Map-based cloning of the *ESP* gene. The *ESP* locus was mapped to the short arm of rice chromosome 1. **b** Expression levels of *Os01g0356951* was determined by semi-quantitative RT-PCR. **c** Expression analysis of *ESP* in the chimeric *Epi-sp* plants. Total RNAs were extracted from 3 independent chimeric plants by separating tissues with different phenotypes. N and sp. indicate RNA extracted from normal and short panicles, respectively. **d** Expression analysis of *ESP* in the progenies of the chimeric *Epi-sp* plants. Total RNAs were extracted from *Epi-normal* and *Epi-sp* plants derived from the chimeric *Epi-sp* plants

To identify the mutated gene responsible for the *Epi-sp* phenotypes, map-based cloning method was employed. Initially, linkage analysis with 250 polymorphic SSR markers spanning a 5–10 cM interval on 12 chromosomes was conducted using a small-scale F_2_ population (80 individuals) derived from a cross between *Epi-sp* and Huajingxian74 (an *indica* rice cultivar). Genetic mapping revealed that the *ESP* locus was associated with one SSR marker RM23 on the short arm of chromosome 1. The *ESP* locus was subsequently narrowed to a region of 5.7 cM between the RM140 and RM446 markers in 290 F_2_ individuals. For fine mapping of *ESP*, insertion-deletion (InDel) markers were designed according to sequence differences between *indica* and *japonica* rice. Ultimately, using a large-scale F_2_ population of 1129 mutant individuals, we mapped *ESP* locus to a 51.2-kb region on chromosome 1 (Fig. 2a). Within this 51.2-kb interval, there are four predicted ORFs (*Os01g0356900*, *Os01g0356951*, *Os01g0357100*, *Os01g0357200*). Genome annotation revealed that *Os01g0356900* encodes a putative extensin-like protein, partial sequences of *Os01g0356951* is similar to flavin monoxygenase-like genes, *Os01g0357100* encodes a putative ferredoxin-nitrite reductase and *Os01g0357200* encodes a sterol-binding domain containing protein. However, we found no nucleotide sequence difference between the mutant and the wild type in this region. We next investigated the expression level of four genes annotated in the mapped region. Expression analysis demonstrated that the transcript level of *Os01g0356951* was dramatically elevated in *Epi-sp* plants (Fig. 2b), while that of three other genes remained unchanged (Additional file 1: Figure S2), compared to wild type plants. Moreover, *Os01g0356951* showed a higher expression in homozygous *Epi-sp* plants than in heterozygous lines (Fig. 2b). These results suggested that the phenotype of *Epi-sp* might be caused by the elevated expression of *Os01g0356951.*

To determine the cause of the metastable phenotype of *Epi-sp*, we performed expression analysis of the *ESP* gene (*Os01g0356951*). Although *ESP* was highly expressed in short panicles of chimeric plants, the *ESP* transcript was not detected in normal panicles of these same plants (Fig. 2c). We also examined *ESP* expression in the progenies of selfed independent chimeric *Epi-sp* plants and found that the *ESP* gene was highly expressed in *Epi-sp* but not in *Epi*-*normal* plants (the progeny from normal panicle of chimeric plant) (Fig. 2d). These results suggest that the epigenetic phenomenon in *Epi-sp* is associated with the misexpression of *ESP* gene.

**Fig. 3:**
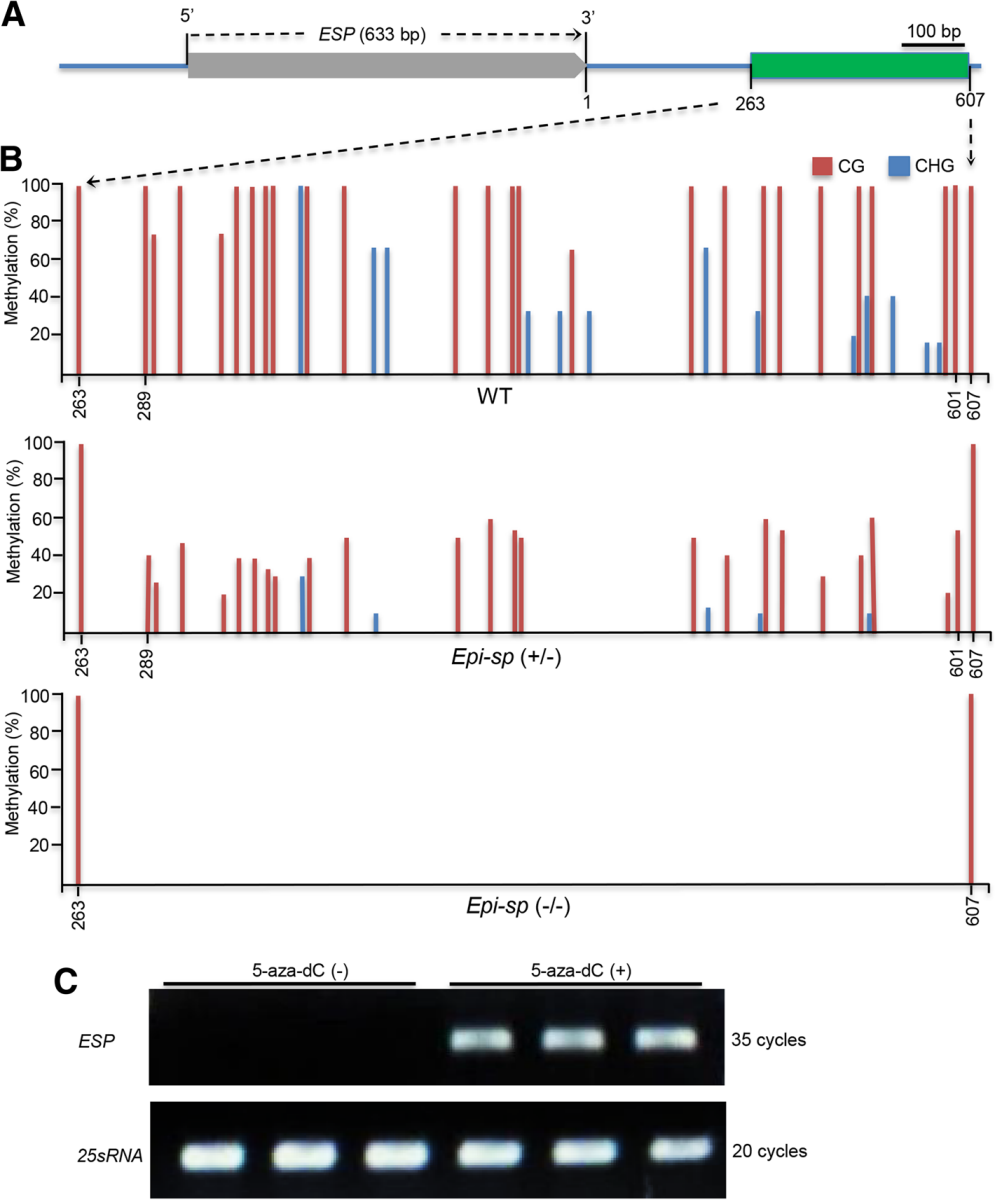
DNA methylation analysis of the *ESP* locus. **a** Schematic representation of *ESP* with methylation locus. Boxes indicate *ESP* (grey) and methylation locus (green). **b** DNA methylation status of bisulfite-sequenced region (as indicated in A) in wild-type (WT) and *Epi-sp* (−/−) plants. Histograms represent the percentage of CG (red) and CNG (blue). **c** Reverse transcription-PCR analyses of *ESP* expression in 7-d-old seedlings treated with (+) or without (−) 5-aza-dC, an inhibitor of DNA methylation. Rice *25sRNA* was used as a control

We found no nucleotide sequence difference between the wild type and *Epi-sp*, but we did observe alteration of *ESP* expression levels in the *Epi-sp* plants, suggesting that the mutation may result from an epigenetic modification. We therefore investigated the DNA methylation status of the *ESP* locus. We performed bisulfite sequencing to analyze their methylation profiles in a 3179-bp genomic region consisting of 633 bp of gene body, 1545 bp of upstream region, and 1001 bp of 3′ distal sequence (Fig. 3a). We observed higher CG and CHG but not CHH DNA methylation in the downstream transcriptional termination region of *ESP* gene in the wild type compared with the *Epi-sp* mutant. All the altered methylation sites occurred in a contiguous 313-bp region at 289 to 601 bp downstream of the transcriptional termination site. This region is hypermethylated in the wild type but is demethylated in the *Epi-sp* mutant, spanning 26 CG sites and 13 CHG sites (Fig. 3b).

To determine whether loss of DNA methylation in the transcriptional termination region (TTR) directly causes the ectopic expression of *ESP* in *Epi-sp*, we treated the wild-type seeds with 5-aza-2′ deoxycytidine (5-aza-dC), an inhibitor of DNA methylation (Chang and Pikaard 2005), to assess its effect on the expression of *ESP*. Transcript levels of *ESP* were measured in 7-d-old seedlings with or without 5-aza-dC treatment (Additional file 1: Figure S3) and found that treatment with 5-aza-dC up-regulated *ESP* expression (Fig. 3c). These results indicated that the DNA methylation in the 3′ end downstream sequence of *ESP* plays an essential role in the regulation of *ESP* expression. Promoter methylation is a general mechanism for suppressing gene expression in eukaryotes. Interestingly, methylation in the transcriptional termination region (TTR) is also highly correlated with gene expression, even higher than that of promoters, especially for CG methylation in rice (Li et al. 2012). In fact, the TTR of *ESP* gene is a CpG island, containing 29 CpG dinucleotide repeats (Additional file 1: Figure S4). Consistently, our study showed that *ESP* TTR hypomethylation caused induced expression of *ESP* in *Epi-sp* plants.

**Fig. 4:**
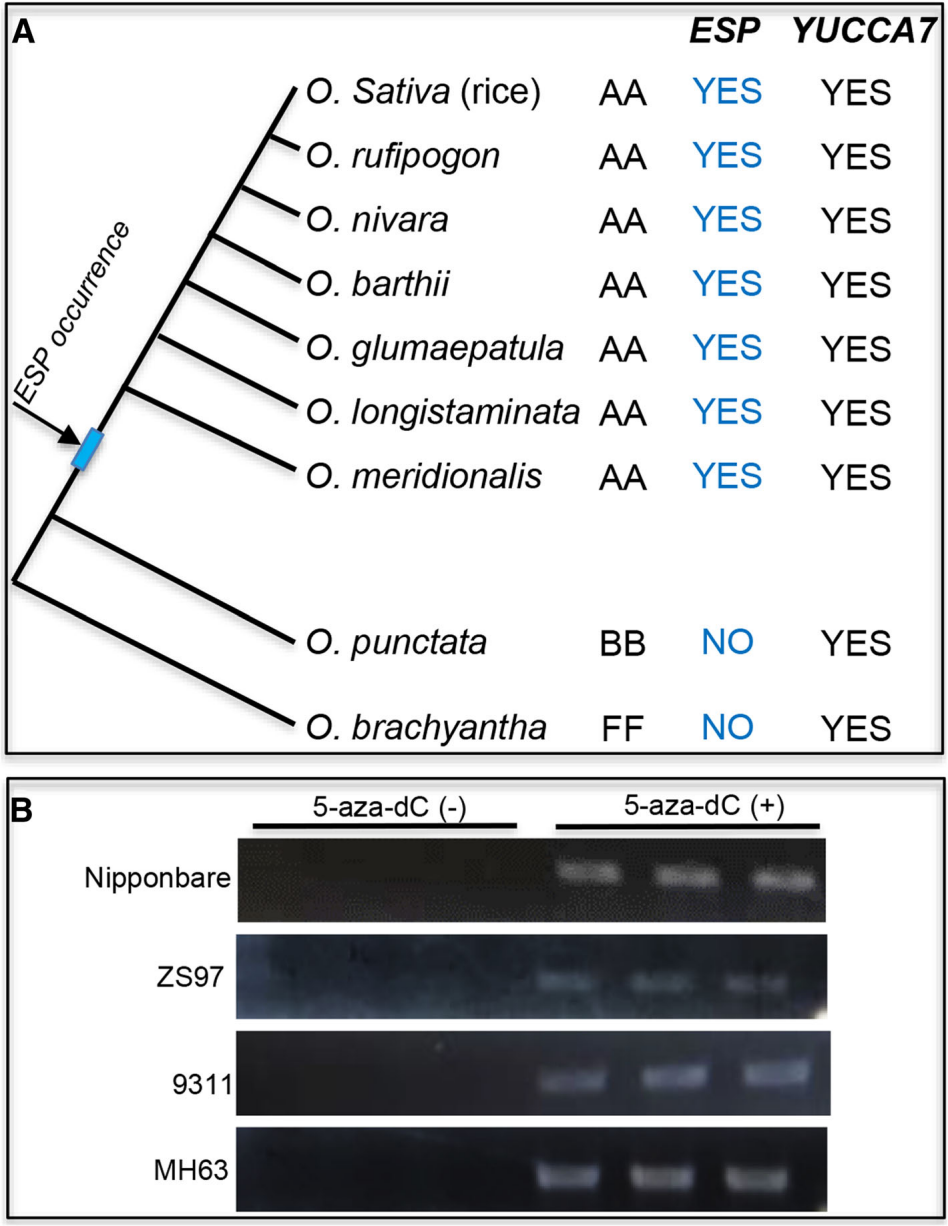
Effect of DNA methylation on *ESP* expression is conserved in cultivated rice. **a** Phylogenetic tree of *ESP* and *YUCCA7* genes. **b** Reverse transcription-PCR analyses of *ESP* expression in 7-d-old seedlings treated with (+) or without (−) 5-aza-dC

To explore the evolutionary significance of the epigenetic regulation of *ESP*, we investigated natural variation in the *ESP* locus and its downstream methylation region (TTR) in 40 accessions of cultivated rice (Additional file 1: Table S1), representing all of the major groups of Asian cultivated rice (Xu et al. 2011). The results showed that the *ESP* locus is conserved in all cultivated rice accessions (data not shown). To further confirm this, we aligned the sequences of the *ESP*, in four known assembled rice genomes, cv Nipponbare, 93–11, MH63 and ZS97, six AA-genome wild rice, one BB-genome (*Oryza punctata*) wild rice and one FF-genome (*Oryza brachyantha*) wild rice. The *ESP* is conserved in the AA-genome species including cultivated rice genomes but is absent in *O. brachyantha* and *Oryza punctata* (Fig. 4a and Additional file 1: Figure S5). Consistent with this, the sequence of the TTR region of *ESP* is also conserved in AA-genome species including cultivated rice (Additional file 1: Figure S6), which might be important for the regulation of *ESP* expression.

To further determine the conserved effect of the DNA methylation on *ESP* expression in cultivated rice accessions, we measured *ESP* transcript levels in four cultivated rice strains (cv MH63, 9311, ZS97 and Nipponbare). Compared with the high expression of *ESP* observed in *Epi-sp*, we observed little or no *ESP* expression in seedlings in all the cultivated rice strains tested (Fig. 4b). Like the pattern of wild-type cv Zhonghua11, the other four cultivated rice accessions also showed higher transcriptional levels of *ESP* in the seedlings treated with 5-aza-dC (Fig. 4b and Additional file 1: Figure S3). Thus, it is tempting to speculate that the occurrence of *ESP* predates the divergence of *indica* and *japonica* rice and the epigenetic regulation of *ESP* expression is evolutionarily conserved in cultivated rice.

**Fig. 5:**
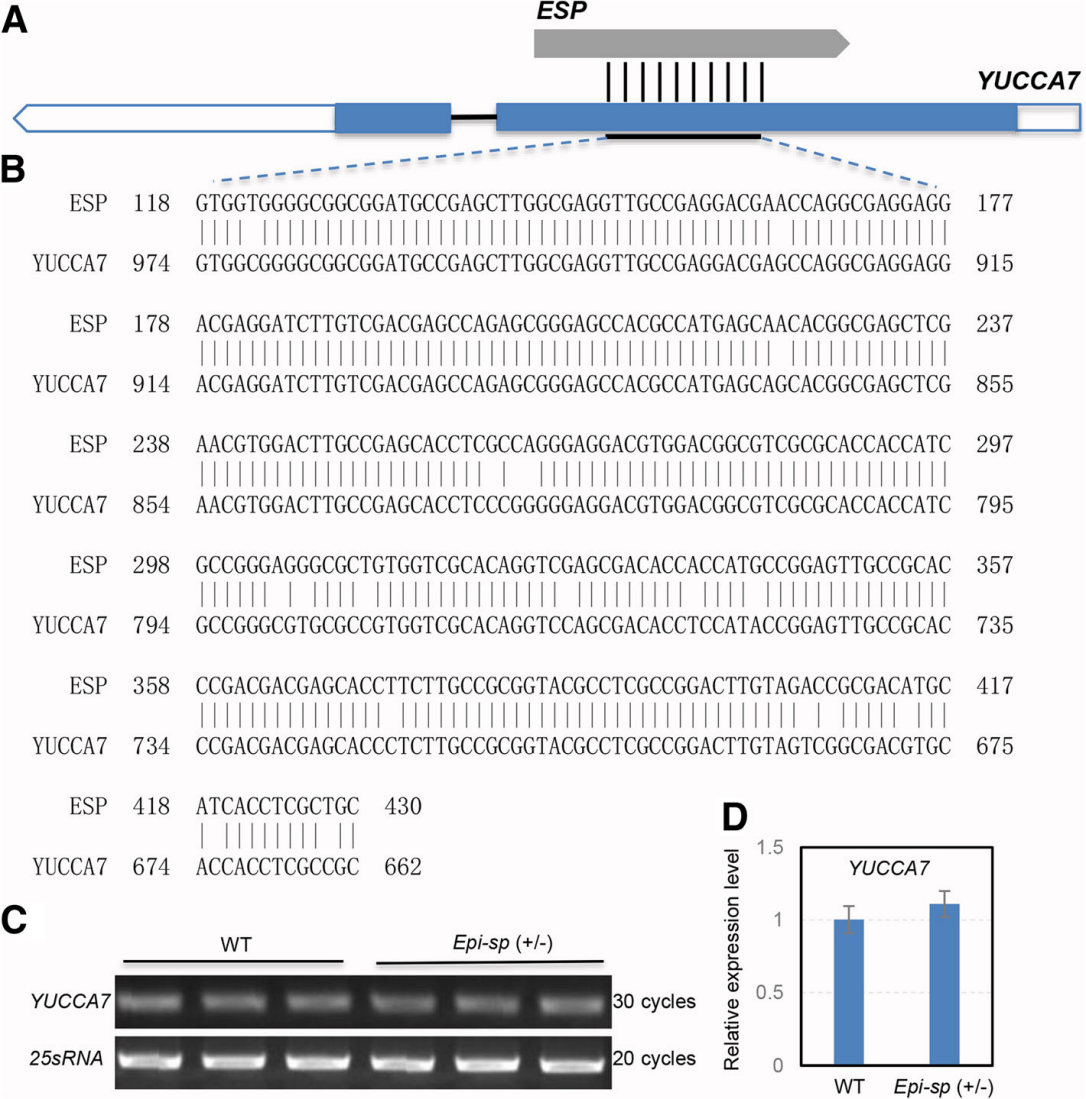
Genome sequence alignment of the *ESP* locus and expression analysis of *YUCCA7*gene. **a** *ESP* had high sequence identity with a putative YUCCA-like gene (*OsYUCCA7*). Blue boxes indicate the putative coding sequence, white boxes indicate the 5′ and 3′ untranslated regions. **b** an alignment of the 313-bp DNA sequence between *ESP* and *YUCCA7*. Expression analysis of *YUCCA7* by reverse transcription-PCR (**c**) and real-time reverse transcription-PCR (**d**) in young panicles (3–5 cm). Rice *25sRNA* was used as a control. Values were means ± SD of three biological replicates

To gain insights into the possible function of the *ESP*, its sequence was used to search rice databases and other public databases. Interestingly, we found that *ESP* had high sequence identity with a putative YUCCA-like gene (*OsYUCCA7*, *Os04g0128900*) (Fig. 5a). However, the similarity between *ESP* and *OsYUCCA7* is only restricted to a reverse-complementing region of about 300-base sequence (Fig. 5b), like a pair of sense/antisense RNA. YUCCAs are a family of important enzymes which catalyze a key rate-limiting step in the tryptophan-dependent pathway for auxin biosynthesis (Mashiguchi et al. 2011; Won et al. 2011). For most *YUCCA* genes of the rice genome, the mean length of coding sequences is more than 1000-bp, encoding about 400 amino acid polypeptides. In addition, we found that three predicted open reading frames (ORFs) of *ESP* shared no sequence similarity with any known protein (Additional file 1: Figure S7). These results suggested that *ESP* might be a long non-coding RNA (lncRNA) gene.

Pseudogenes are usually characterized by a combination of homology to a known gene and loss of some functionality (Mighell et al. 2000). Compared to *YUCCA7* gene, *ESP* shares very high sequence identity with *OsYUCCA7*, but has extremely low protein coding probability and gene expression level in cultivated rice (Fig. 4b). Phylogenetic analyses revealed that *YUCCA7*-like genes are well evolutionally conserved in AA-, BB-, and FF-genome wild rice, but *ESP* is absent in BB-genome (*Oryza punctata*) and FF-genome (*Oryza brachyantha*) wild rice (Fig. 4a). Thus, it is highly likely that *ESP* is the pseudogene of *YUCCA7* gene. Based on the findings mentioned above, we proposed that the *ESP* might be a pseudogene-derived antisense lncRNA gene.

Despite pseudogenes being considered as nonfunctional genomic locus, some pseudogenes may be functional, similar to other kinds of noncoding DNA, which can perform regulatory functions to their parent genes (Mighell et al. 2000). To explore the effect of *ESP* on its parent gene, we assessed the relationship between *ESP* and *OsYUCCA7* expression in young panicle (3-5 cm) by qRT-PCR. Expression analysis revealed that the expression level of *OsYUCCA7* in heterozygous *Epi-sp (+/−)* mutant was similar to that in WT (Fig. 5c, d). The result suggested that *ESP* overexpression probably had little effect on *OsYUCCA7* transcription. However, whether *ESP* regulates the level of OsYUCCA7 protein production remains to be elucidated.

In this study, *Epi-sp* mutant shows low levels of DNA CG and CHG methylation in the TTR of *ESP* gene (Fig. 3b), causing ectopic *ESP* expression (Fig. 2b) and a dense and short panicle architecture in rice (Fig. 1). It is important to point out that, although the mechanism for the spontaneous hypomethylation of *ESP* TTR remains unknown, the TTR of *ESP* gene displays the characteristic of CpG island (Additional file 1: Figure S4). Since there is no CHH methylation in the TTR of *ESP* (Fig. 3b), CG and CHG hypermethylation in this region is likely not established by the RNA-directed DNA methylation (RdDM) pathway. Consequently, hypomethylation of this region in the *Epi-sp* mutant is probably not due to loss of small interfering RNAs (siRNAs). Due to the mechanistic nature of maintenance methyltransferases, TTR hypomethylation is also likely not established by lack of OsMET1 and OsCMT3 activities in a locus-specific manner. Therefore, we speculate the epiallele is probably induced by some sort of aberrant active DNA demethylation activity.

In this study, we report the identification and characterization of the rice *Epi-sp* mutant that displays dense and short panicle phenotype. Morphological analysis showed that the dense and short panicle phenotype in *Epi-sp* mutants resulted from the decrease in the length of the rachis, primary and secondary branches. The map-based cloning of *ESP* revealed that it encodes a putative pseudogene-derived lncRNA. Further studies to clarify the molecular mechanism of *ESP* should help shed light on the developmental processes underlying the formation of panicle in rice.

## Additional files

Additional file 1: **Figure S1.** Panicle morphology of *Epi-sp* mutant. **Figure S2.** Expression analysis of three genes annotated in the mapped region in the wild-type and *esp* mutant plants. **Figure S3.** Phenotype of 10-day-old seedlings treated with (+) or without (−) 5-aza-dC. **Figure S4.** Predicted CpG island in the TTR of *ESP*. **Figure S5.** Multiple sequence alignment of the *ESP* gene. **Figure S6.** Multiple sequence alignment for the TTR region of *ESP*. **Figure S7.** Open reading frames (ORFs) prediction of *ESP* gene. **Table S1.** 40 accessions of cultivated line. **Table S2.** Primers used in this study. **File S1.** Experimental procedures (PDF 638 kb)

## Abbreviations

5-aza-dC: 5-aza-2′ deoxycytidine
ESP: Epigenetic Short Panicle
lncRNA: long noncoding RNA
SAM: shoot apical meristem
TTR: transcriptional termination region
