# Supplementary material for: Epigenetic modification of ESP, encoding a putative long noncoding RNA, affects panicle architecture in rice
Source: Rice (N Y). 2019 Apr 3;12:20. doi: 10.1186/s12284-019-0282-1 (PMC6447638; doi:10.1186/s12284-019-0282-1)
Supplement: Supplementary file 1 — Figure S1. Panicle morphology of Epi-sp mutant. Figure S2. Expression analysis of three genes annotated in the mapped region in the wild-type and esp mutant plants. Figure S3. Phenotype of 10-day-old seedlings treated with (+) or without (−) 5-aza-dC. Figure S4. Predicted CpG island in the TTR of ESP. Figure S5. Multiple sequence alignment of the ESP gene. Figure S6. Multiple sequence alignment for the TTR region of ESP. Figure S7. Open reading frames (ORFs) prediction of ESP gene. Table S1. 40 accessions of cultivated line. Table S2. Primers used in this study. File S1. Experimental procedures (PDF 638 kb) [file 12284_2019_282_MOESM1_ESM.pdf]

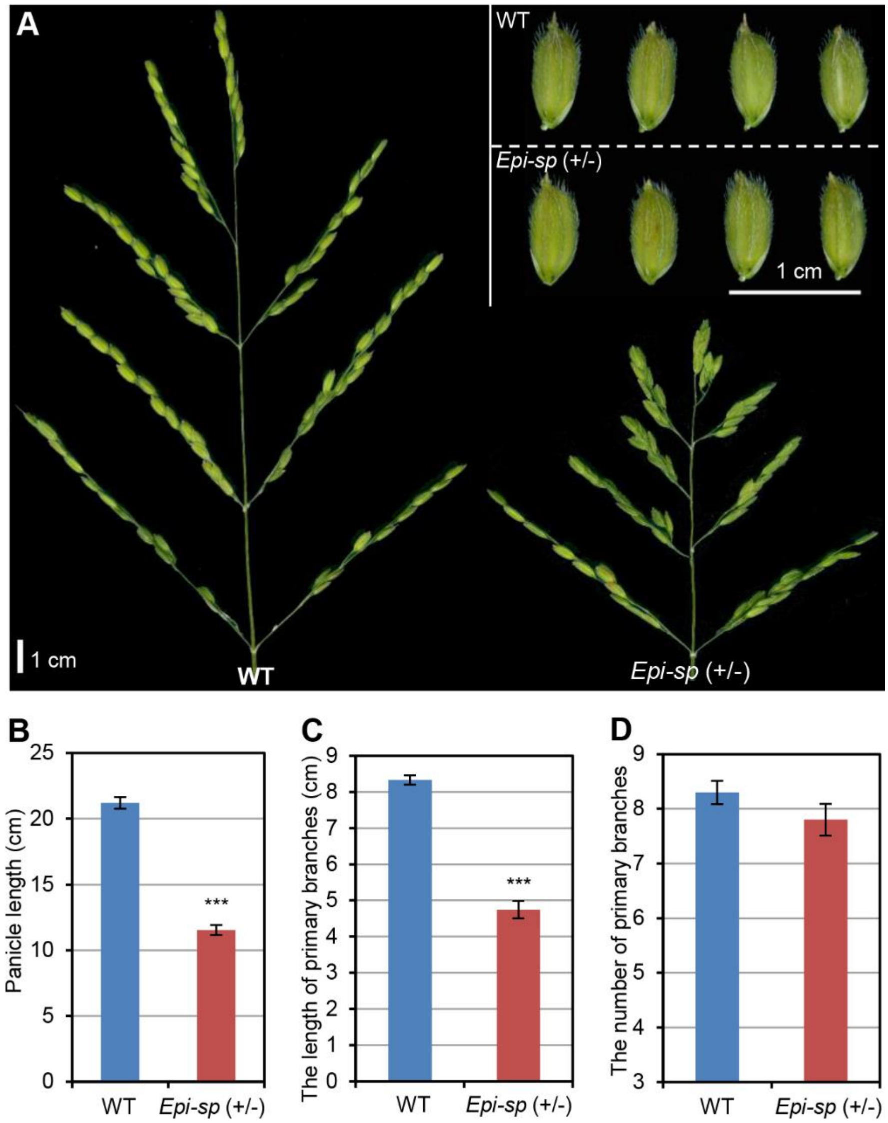

Fig. S1 Panicle morphology of *Epi-sp* mutant. **a** Comparison of the panicle branching and grains between the wild type and *Epi-sp* mutant. Panicle length (**b**), the length of primary branches (**c**) and number of primary branches per panicle (**d**) of wild type and *Epi-sp*. Data are averages of 15 plants (±SD). Asterisks indicate the significance of differences between wild type and *Epi-sp* plants, as determined by Student's t-test: *** $P < 0.001$.

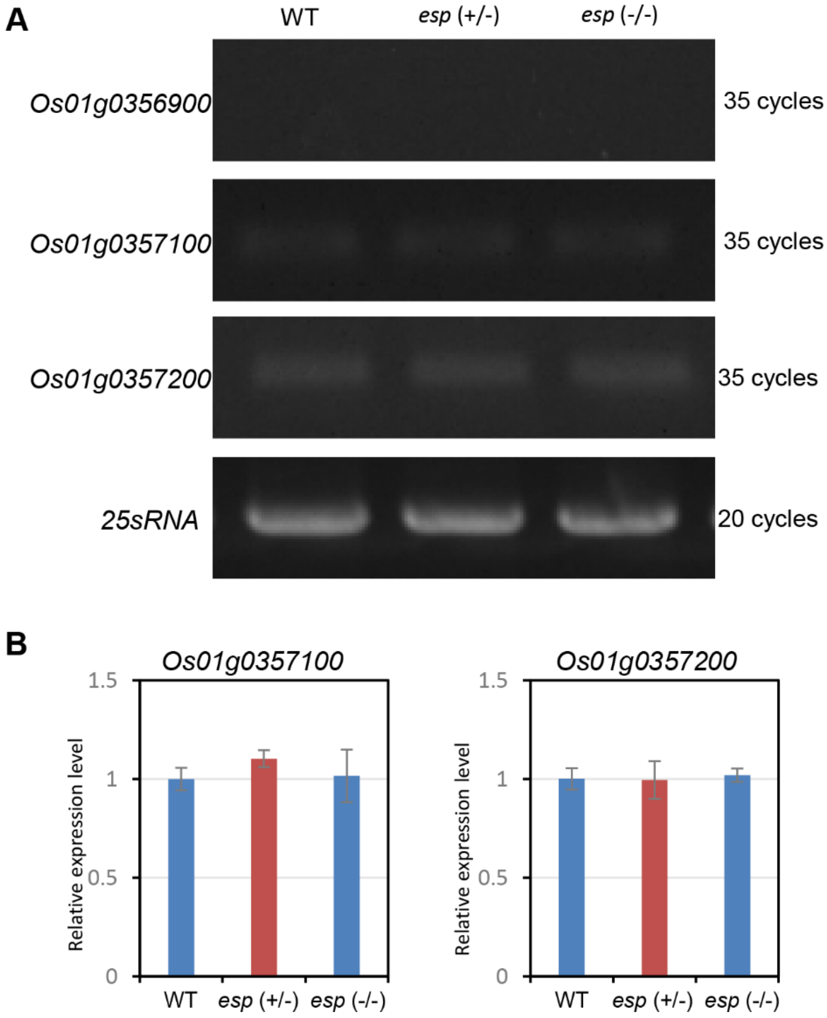

Fig. S2 Expression analysis of three genes annotated in the mapped region in the wild-type and *esp* mutant plants. **a** Semi-quantitative RT-PCR analysis of three candidate genes (*Os01g0356900*, *Os01g0357100* and *Os01g0357200*) in the wild-type and *esp* mutant plants. Transcripts of *Os01g0356900* gene was almost undetectable in the wild-type and *esp* mutant plants. **b** qRT-PCR analysis of *Os01g0357100* and *Os01g0357200* with detectable expression in (a)**.** Values are means ±SD of three biological replicates. Total RNA was isolated from the young panicle of the heterozygous *Epi-sp* (+/-) mutant or shoot apical meristem of the homozygous *Epi-sp* (-/-) mutant at heading stage. Rice *25sRNA* was used as a control.

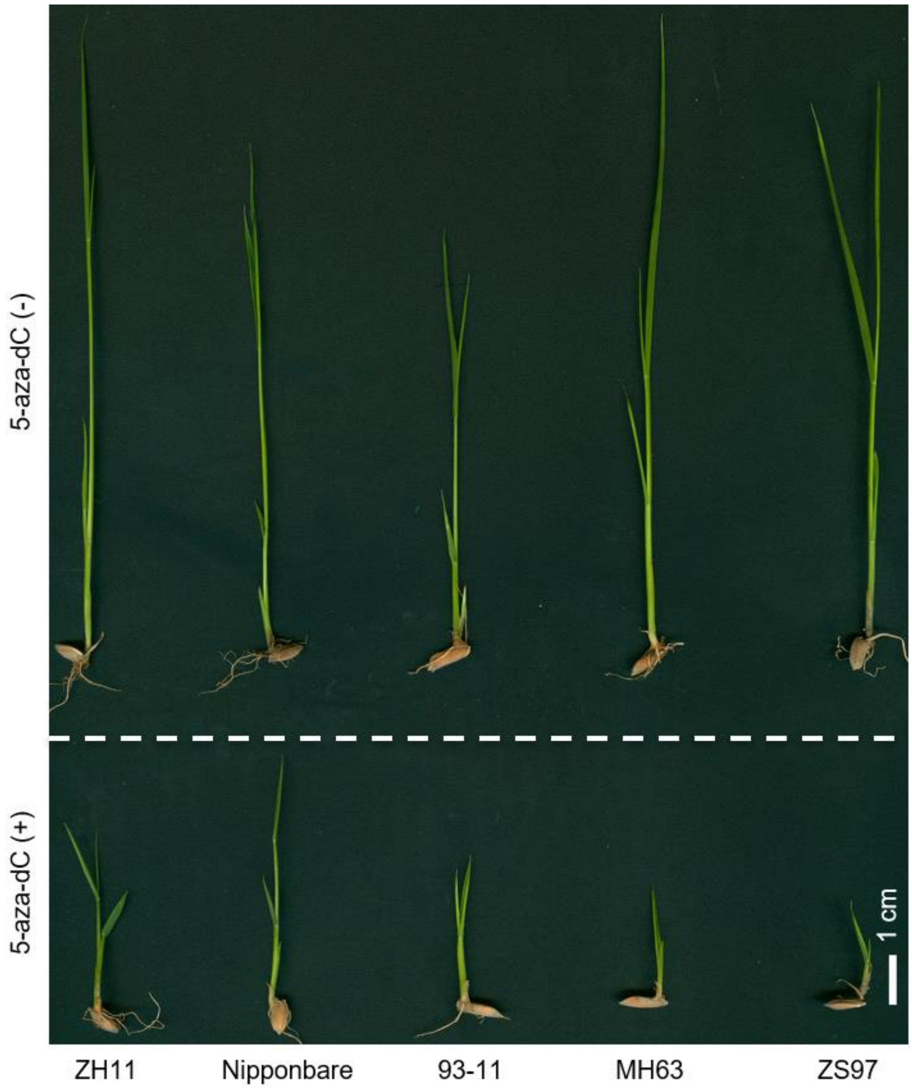

Fig. S3 Phenotype of 10-day-old seedlings treated with (+) or without (–) 5-aza-dC. Rice seeds were soaked in water at 30°C for 24 h. The seeds were then immersed in 20 mM Tris-HCl, pH 7.5, with or without 0.3 mM 5-aza-dC (A3656; Sigma) at 28°C for 72 h in the dark. After washing, seeds were planted in soil. Ten-day-old seedlings were taken photos.

CGTTGATAAGTCACTACTACAACCATCGGCGACCTTTCTCGGGATCCAAGCATGTCGACC

CCGCCAACGTGGCGTCGGTGCAGGGCACCGAGATGAACACCACGGGGCTATTTGCCTGTC

CAGGGTCATCCTAGGCTTAAGGCCACGACACTCAAGGACGTGGTGGGCGGCGTCACAGAG

GTGCTCCCAGCGAACAAGCTGGCCACCAAGGAGGACGCCGACAAGGTGGCGGCCACCGCT

ATGCAGAAACGATGGGAGGCATGCCGGTGACGACAAGGAGTTAACACGATCCATTTAGTC

CCGATCCGAGTTTATCAGGAATTCAATCCTGCACCGTGCGGTTACG

Fig. S4 Predicted CpG island in the TTR of *ESP*. The TTR of *ESP* gene contains a large number of CpG dinucleotide repeats. The CpG island is identified by three primary characteristics: it is more than 200 bp long (313 bp), have over 50% GC composition (58.4%), and retain an observed/expected ratio of CpG dinucleotides greater than 0.6.

```
O.meridionalis      AGATTG----AAAAAATCGCCATGTCACCGAGTCGCCAGTCGCCGCCTCGACGCCGGTCACCGCCTCGTCGCCTCGACGCAGCCCACCGCCGGCGCTGCACGCCCCCCTCCAAGCCGTCG
O.longistaminata    AGATCGAAAAAAAAAATCGACATCTCGCTGCCTCGCTGGT-------TCGATGCTAGTCGCCG--------CCTCAACGCAGCCCACCGCCGGCGCTGCACGCCCCCCTCCAAGCCGTCG
O.barthii           AGATCG----AAAAAATCGCCATCTCGCCGAGTTGCCAGTCGCCGCCTTGACGCCGGTCGCCA---------------------TACCGCCGGCGCTGCACGCCCCCCTCCAAGCCATCG
O.glumaepatula      AGATCA----AAAAAATCGCCATCTCGCCGAGACACCAGTCGCCGCCTCGACGCCGGTCGCCG---------------------TACCGCCGGCGCTGCACGCTCCCCTCCAAGCCGTCG
MH63                AGATCG----AAAAAATTGCCATCTCACCGAGTCGCCAGTCGCCGCCTCGACGCCGGTCGCCG---------------------TACCGCCGGCGCTGCACGCCCCCCTCCAAGCCGTCG
R498                AGATCG----AAAAAATTGCCATCTCACCGAGTCGCCAGTCGCCGCCTCGACGCCGGTCGCCG---------------------TACCGCCGGCGCTGCACGCCCCCCTCCAAGCCGTCG
Zhonghua11          AGATCG----AAAAAATCGCTATCTCGCCGAGTCGCCAGTCACCGCCTCGACGCCGGTCGCCG---------------------TACCGCCGGCGCTGCACGCCCCCCTCCAAGCCGTCG
Nipponbare          AGATCG----AAAAAATCGCTATCTCGCCGAGTCGCCAGTCACCGCCTCGACGCCGGTCGCCG---------------------TACCGCCGGCGCTGCACGCCCCCCTCCAAGCCGTCG
O.rufipogon         AGATCG----AAAAAATCGCTATCTCGCCGAGTCGCCAGTCACCGCCTCGACGCCGGTCGCCG---------------------TACCGCCGGCGCTGCACGCCCCCCTCCAAGCCGTCG
ZS97                AGATCG----AAAAAATCGCCATCTCGCCGAGTCGCCAGTCGCCGCCTCAACGCCGGTCGCCG---------------------TACCGCCGGCGCTGCACGCCCCCCTCCAAGCCGTCG
9311                AGATCG----AAAAAATCGCCATCTCGCCGAGTCGCCAGTCGCCGCCTCAACGCCGGTCGCCG---------------------TACCGCCGGCGCTGCACGCCCCCCTCCAAGCCGTCG
O.nivara            AGATCG----AAAAAATCGCCATCTCGCCGAGTCGCCAGTCGCCGCCTCAACGCCGGTCGCCG---------------------TACCGCCGGCGCTGCACGCCCCCCTCCAAGCCGTCG
                    ****      ******* *  ** ** * *     *  **       *  * **  *** **                       ****************** ************ ***

O.meridionalis      CCCCGTCGCCCCCAGCCGCCCCTGCGGGCCGGTGGTGG----------------------------------------------------------------------------------
O.longistaminata    CCCCGTCGCCCCCAGCCGCCCCAGCAGGCCGGTGGTGGGGCGGCGGATGCCGAGCTTGGTGAGGTTGCCGAGGACGAACCAGGCGAGGAGGACGAGGATCTTGTCGACGAGCCAGAGCGG
O.barthii           CCCTGTCGCCCCCAGCCGC---------CCGGTGGTGGGGCGGCGGATGCCGAGCTTGGCGAGGTTGCCGAGGACGAACCAGGCGAGGAGGACGAGGATCTTGTCGACGAGCCAGAGCGG
O.glumaepatula      CCCCGTCGCCC-------------------GGTGGTGGGGCGGCGGATGCCGAGCTTGGCGAGGTTGCCGAGGACGAACCAGGCGAGGAAGACGAGGATCTTGTCGACGAGCCAGAGCGG
MH63                CCCCATCGCCCCCAGCCGC---------CCGGTGGTGGGGCGGCGGATGCCGAGCTTGGCGAGGTTGCCAAGGACGAACCAGGCGAGGAGGACGAGGATCTTGTCGACGAGCCAGAGCGG
R498                CCCCATCGCCCCCAGCCGC---------CCGGTGGTGGGGCGGCGGATGCCGAGCTTGGCGAGGTTGCCAAGGACGAACCAGGCGAGGAGGACGAGGATCTTGTCGACGAGCCAGAGCGG
Zhonghua11          CCCCATCGCCCCCAGCCGC---------CCAGTGGTGGGGCGGCGGATGCCGAGCTTGGCGAGGTTGCCGAGGACGAACCAGGCGAGGAGGACGAGGATCTTGTCGACGAGCCAGAGCGG
Nipponbare          CCCCATCGCCCCCAGCCGC---------CCAGTGGTGGGGCGGCGGATGCCGAGCTTGGCGAGGTTGCCGAGGACGAACCAGGCGAGGAGGACGAGGATCTTGTCGACGAGCCAGAGCGG
O.rufipogon         CCCCATCGCCCCCAGCCGC---------CCAGTGGTGGGGCGGCGGATGCCGAGCTTGGCGAGGTTGCCGAGGACGAACCAGGCGAGGAGGACGAGGATCTTGTCGACGAGCCAGAGCGG
ZS97                CCCCATCGCCCCCAGCCGC---------CTGGTGGTGGGGCAGCGGATGCCGAGCTTGGCGAGGTTGCCGAGGACGAACCAGGCGAGGAGGACGAGGATCTTGTCGACGAGCCAGAGCGG
9311                CCCCATCGCCCCCAGCCGC---------CTGGTGGTGGGGCAGCGGATGCCGAGCTTGGCGAGGTTGCCGAGGACGAACCAGGCGAGGAGGACGAGGATCTTGTCGACGAGCCAGAGCGG
O.nivara            CCCCATCGCCCCCAGCCGC---------CCGGTGGTGGGGCAGCGGATGCCGAGCTTGGCGAGGTTGCCGAGGACGAACCAGGCGAGGAGGACGAGGATCTTGTCGACGAGCCAGAGCGG
                    ***  ******                    *******

O.meridionalis      --------------------------------------------CGACCACCTCGCCAGGGA-GGACGTGGACGGCGTCGCGTACCACCATCGCCGGGCGGGCGCTGTGGTCGCACAGGT
O.longistaminata    GAGCCACGCCATGAGCAACACGGCGAGCTCGAACGTGGACTTGCCGAGCACCTCGCTAGGGA-GGACGTGGACGGCGTCGCGCACCCCCATCGCCGGGAGGGCGCTGTGGTCGAACAGGT
O.barthii           AAGCCACGCCATGAGCAACACGGCGAGCTCGAACGTGGACTTGCCGAGCACCTCGCCAGGGAGGGACGTGGACGGCGTCGCGCACCACCATCGCCGGGAGGGCGCTGTGGTCGCACAGGT
O.glumaepatula      GAGCCACGCCATGAGCAACACGGCGAGCTCGAACGTGGACTTGCCGAGCACCTCGCCAGGGA-GGACGTGGACGGCGTCGCGCACCACCATCGCCGGGAGGGCGCTGTGGTCGCACAGGT
MH63                GAGCCACGCCATGAGCAACACGGCGAGCTCGAACGTGGACTTGCCGAGCACCTCGCCAGGGA-GGACGTGGACGGCGTCGCGCACCACCATTGCCGGGAGGGCGCTGTGGTCGCACAGGT
R498                GAGCCACGCCATGAGCAACACGGCGAGCTCGAACGTGGACTTGCCGAGCACCTCGCCAGGGA-GGACGTGGACGGCGTCGCGCACCACCATTGCCGGGAGGGCGCTGTGGTCGCACAGGT
Zhonghua11          GAGCCACGCCATGAGCAACACGGCGAGCTCGAACGTGGACTTGCCGAGCACCTCGCCAGGGA-GGACGTGGACGGCGTCGCGCACCACCATCACCGGGAGGGCGCTGTGGTCGCACAGGT
Nipponbare          GAGCCACGCCATGAGCAACACGGCGAGCTCGAACGTGGACTTGCCGAGCACCTCGCCAGGGA-GGACGTGGACGGCGTCGCGCACCACCATCGCCGGGAGGGCGCTGTGGTCGCACAGGT
O.rufipogon         GAGCCACGCCATGAGCAACACGGCGAGCTCGAACGTGGACTTGCCGAGCACCTCGCCAGGGA-GGACGTGGACGGCGTCGCGCACCACCATCGCCGGGAGGGCGCTGTGGTCGCACAGGT
ZS97                GAGCCACGCCATGAGCAACACGGCGAGCTCGAACGTGGACTTGCCGAGCACCTCGCCAGGGA-GGACGTGGACGGCGTCGCGCACCACCATCGCCGGGAGGGCGCTGTGGTCGCACAGGT
9311                GAGCCACGCCATGAGCAACACGGCGAGCTCGAACGTGGACTTGCCGAGCACCTCGCCAGGGA-GGACGTGGACGGCGTCGCGCACCACCATCGCCGGGAGGGCGCTGTGGTCGCACAGGT
O.nivara            GAGCCACGCCATGAGCAACACGGCGAGCTCGAACGTGGACTTGCCGAGCACCTCGCCAGGGA-GGACGTGGACGGCGTCGCGCACCACCATCGCCGGGAGGGCGCTGTGGTCGCAGAGGT
                                                                *** ******** ***** ******************* *** ****  ***** ************** * ****
O.meridionalis      CGAGCGACACCTCCATGCCGGAGTTGCCGCACCCGACGACGAGCACCTTCTTGCTGCGGTACGCCTCGCCGGACTTGTAGTCCGCGACATGCATCACCTCGCTGCTATATTTGTTCTTGG
O.longistaminata    CGAGCGACACCACCATGCCGGAGTTGCCGCACCCGACGACGAGCACCTTCTTGCTGCGGTACGCCTCGCCGGACTTGTAGACCGCGACATGCATCACCTCCCTGCTATATTTGTTCTTGG
O.barthii           CGAGCGACACCACCATGCCGGAGTTGCCGCACCCGACGACGAGCACCTTCTTGCCGCGGTACGCCTCGCCGGACTTGTAGACCGCGACATGCATCACCTCGCTGCTATATTTGTTCTTGG
O.glumaepatula      CGAGCGACACCACCATGCCGGAGTTGCCGCACCCGACGACGAGCACCTTCTTGCCGCGGTACGCCTCGCCGGACTTGTAGACCGCGACATGCATCACCTCGCTGCTATATTTGTTCTTGG
MH63                CGAGCGACACCACCATGCCGGAGTTGCCGCACCCGACGACGAGCACCTTCTTGCCGCGGTACGCCTCGCCGGACTTGTAGACCGCGACATGCATCACCTCGCTGCTATATTTGTTCTTGG
R498                CGAGCGACACCACCATGCCGGAGTTGCCGCACCCGACGACGAGCACCTTCTTGCCGCGGTACGCCTCGCCGGACTTGTAGACCGCGACATGCATCACCTCGCTGCTATATTTGTTCTTGG
Zhonghua11          CGAGCGACACCACCATGCCGGAGTTGCCGCACCCGACGACGAGCACCTTCTTGCCGCGGTACGCCTCGCCGGACTTGTAGACCGCGACATGCATCACCTCGCTGCTATATTTGTTCTTGG
Nipponbare          CGAGCGACACCACCATGCCGGAGTTGCCGCACCCGACGACGAGCACCTTCTTGCCGCGGTACGCCTCGCCGGACTTGTAGACCGCGACATGCATCACCTCGCTGCTATATTTGTTCTTGG
O.rufipogon         CGAGCGACACCACCATGCCGGAGTTGCCGCACCCGACGACGAGCACCTTCTTGCCGCGGTACGCCTCGCCGGACTTGTAGACCGCGACATGCATCACCTCGCTGCTATATTTGTTCTTGG
ZS97                CGAGCGACACCACCATGCCGGAGTTGCCACACCCGACGACGAGCACCTTCTTGCCGCGGTACGCCTCGCCGGACTTGTAGACCGCGACATGCATCACCTCGCTGCTATATTTGTTCTTGG
9311                CGAGCGACACCACCATGCCGGAGTTGCCACACCCGACGACGAGCACCTTCTTGCCGCGGTACGCCTCGCCGGACTTGTAGACCGCGACATGCATCACCTCGCTGCTATATTTGTTCTTGG
O.nivara            CGAGCGACACCACCATGCCGGAGTTGCCGCACCCGACGACGAGCACCTTCTTGCCGCGGTACGCCTCGCCGGACTTGTAGACCGCGACATGCATCACCTCGCTGCTATATTTGTTCTTGG
                    *********** **************** ************************* ************************* ******************* *******************

O.meridionalis      ACTGTTGAAACTTGCTGTCAGTGGGTGTGTTCAGAATTGCTGCTGCAGCTTGTAGCGAATTTGTAATGCAGCAGCTGCAGCTTGTATGGCTGCCGAGCAGAGCGAGTGTTGCTATCTG-T
O.longistaminata    ACTGTGGAGACTTGCTGTCAGTGGGTGTGTTCAGAATTGCTGCTGCAGCTTGCAGCGAATTTGTGATGCAGCAGCTGCAGCTTGTATGGCTGCCGAGCAGAGCGAGTGTTGCTATCTG-T
O.barthii           ACTGTGGAGACTTGCTGTCAGTGGGTGTGTTCAGAATTGCTGCTGCAGCTTGCAGCGAATTTGTGATGCAGCAGCTGCAGCTTGTATGGCTGCCGAGCAGAGCGAGTGTTGCTATCTG-T
O.glumaepatula      ACTGTGGAGACTTGCTGTCAGTGGGTGTGTTCAGAATTGCTGCTGCAGCTTGCAGCGAATTTGTGATGCAGCAGCTGCAGCTTGTATGGCTGCCGAGCAGAGCGAGTGTTGCTATCTG-T
MH63                ACCGTGGAGACTTGCTGTCAGTGGGTGTGTTCAGAATTGCTGCTGCAGCTTGCAGCGAATTTGTGATGCAGCAGCTACAGCTTGTATGGCTGCCGAGTAGAGCGAGTGTTGCTATCTGTT
R498                ACCGTGGAGACTTGCTGTCAGTGGGTGTGTTCAGAATTGCTGCTGCAGCTTGCAGCGAATTTGTGATGCAGCAGCTACAGCTTGTATGGCTGCCGAGTAGAGCGAGTGTTGCTATCTGTT
Zhonghua11          ACTGTGGAGACTTGCTGTCAGTGGGTGTGTTCAGAATTGCTGCTGCAGCTTGCAGCGAATTTGTGATGCAGCAGCTGCAGCTTGTATGGCTGCCGAGTAGAGCGAGTGTTGCTATCTG-T
Nipponbare          ACTGTGGAGACTTGCTGTCAGTGGGTGTGTTCAGAATTGCTGCTGCAGCTTGCAGCGAATTTGTGATGCAGCAGCTGCAGCTTGTATGGCTGCCGAGTAGAGCGAGTGTTGCTATCTG-T
O.rufipogon         ACTGTGGAGACTTGCTGTCAGTGGGTGTGTTCAGAATTGCTGCTGCAGCTTGCAGCGAATTTGTGATGCAGCAGCTGCAGCTTGTATGGCTGCCGAGTAGAGCGAGTGTTGCTATCTG-T
ZS97                ACTGTGGAGACTTGCTGTCAGTGGGTGTGTTCAGAATTGCTGCTGCAGCTTGCAGCGAATTTGTGATGCAGCAGCTACAGCTTGTATGGCTGCCGAGTAGAGCGAGTGTTGCTATCTGTT
9311                ACTGTGGAGACTTGCTGTCAGTGGGTGTGTTCAGAATTGCTGCTGCAGCTTGCAGCGAATTTGTGATGCAGCAGCTACAGCTTGTATGGCTGCCGAGTAGAGCGAGTGTTGCTATCTGTT
O.nivara            ACTGTGGAGACTTGCTGTCAGTGGGTGTGTTCAGAATTGCTGCTGCAGCTTGCAGCGAATTTGTGATGCAGCAGCTACAGCTTGTATGGCTGCCGAGTAGAGCGAGTGTTGCTATCTGTT
                    ** ** ** ******************************************* *********** *********** ******************** ******************** *

O.meridionalis      TTTTGTTCTCTTTTTTAGAAATTTCGCCCGCAAATTTTAAATTTGAATTCAAATTTTTAAAAGAACTAG
O.longistaminata    TTTTGTTCTCTTTTTCAGAAATTTCGCCCGCAAATTTTAAATTTGAATTCAAATTTTTAAAAGAACTAG
O.barthii           TTTTGTTCTCTTTTTCAGAAATTTCGCCCGCAAATTTTAAATTTGAATTCAAATTTTTAAAAGAACTAG
O.glumaepatula      TTTTGTTCTCTTTTTCAGAAATTTCGCCCGCAAATTTTAAATTTGAATTCAAATTTTTAAAAGAACTAG
MH63                TTTTGTTCTCTTTTTCAGAAATTTCGCCCGCAAATTTTAAATTTGAATTCAAATTTTTAAAAGAACTAG
R498                TTTTGTTCTCTTTTTCAGAAATTTCGCCCGCAAATTTTAAATTTGAATTCAAATTTTTAAAAGAACTAG
Zhonghua11          TTTTGTTCTCTTTTTCAGAAATTTCGCCCGCAAATTTTAAATTTGAATTCAAATTTTTAAAAGAACTAG
Nipponbare          TTTTGTTCTCTTTTTCAGAAATTTCGCCCGCAAATTTTAAATTTGAATTCAAATTTTTAAAAGAACTAG
O.rufipogon         TTTTGTTCTCTTTTTCAGAAATTTCGCCCGCAAATTTTAAATTTGAATTCAAATTTTTAAAAGAACTAG
ZS97                TTTTGTTCTCTTTTTCAGAAATTTCGCCCGCAAATTTTAAATTTGAATTCAAATTTTTAAAAGAACTAG
9311                TTTTGTTCTCTTTTTCAGAAATTTCGCCCGCAAATTTTAAATTTGAATTCAAATTTTTAAAAGAACTAG
O.nivara            TTTTGTTCTCTTTTTCAGAAATTTCGCCCGCAAATTTTAAATTTGAATTCAAATTTTTAAAAGAACTAG
                    *************** *****************************************************
```

Fig. S5 Multiple sequence alignment of the *ESP* gene.

```
Zhonghua11          CGTTGATAAGTCACTACTACAACCATCGGCGACCTTTCTCGGGATCCAAGCATGTCGACC
Nipponbare          CGTTGATAAGTCACTACTACAACCATCGGCGACCTTTCTCGGGATCCAAGCATGTCGACC
9311                CGTTGATAAGTCACTACTACAACCATCGGCGACCTTTCTCGGGATCCAAGCATGTCGACC
MH63                CGTTGATAAGTCACTACTACAACCATCGGCGACCTTTCTCGGGATCCAAGCATGTCGACC
R498                CGTTGATAAGTCACTACTACAACCATCGGCGACCTTTCTCGGGATCCAAGCATGTCGACC
ZS97                CGTTGATAAGTCACTACCATAACCATCGGCGACCTTTCTCGGGATCCAAGCATGTTGACC
O.meridionalis      -GTTGATAAGTCACTACTACAACCATCGGCGACCTTTCTCGGGATCCAAGCATGTCGACC
O.glumaepatula      --TTGATAAGTCACTACTACAACCATCGGCGACCTTTCTCGGGATCCAAGCATGTCGACC
O.barthii           CGTTGATAAGTCACTACTACAACCATTGGCGACCTTTCTCGGGATCCAAGCATGTCGACC
O.rufipogon         CGTTGATAAGTCACTACTACAACCATCGGCGACCTTTCTCGGGATCCAAGCATGTCGACC
O.nivara            CGTTGATAAGTCACTACCACAACCATCGGCGACCTTTCTCGGGATCCAAGCATGTTGACC
O.longistaminata    CGTTGATAAGTCACTACTACAACCATCGGCGACCTTTCTCGGGATCCAAGCATGTCGACC
                      *************** * ****** **************************** ****

Zhonghua11          CCGCCAACGTGGCGTCGGTGCAGGGCACCGAGATGAACACCACGGGGCTATTTGCCTGTC
Nipponbare          CCGCCAACGTGGCGTCGGTGCAGGGCACCGAGATGAACACCACGGGGCTATTTGCCTGTC
9311                CCGCCAACGTGGCGTCGGTGCAGGGCACCGAGATGAACACCACGGGGCTATTTGCCTGTC
MH63                TCGCCAACGTGGCGTCGGTGCAGGGCACCGAGATGAACACCACGGGGCTATTTGCCTGTC
R498                TCGCCAACGTGGCGTCGGTGCAGGGCACCGAGATGAACACCACGGGGCTATTTGCCTGTC
ZS97                CCGCCAACGTGGCGTCGGTGCAGGGCACCGAGATGAACACCACGGGGCTATGTGCCTGTC
O.meridionalis      CCGCCAACGTGGCGTCGGTGCAGGGCACCGAGATGAGTACCATGGGGCTATTTGCCTGTC
O.glumaepatula      CCGCCAACATGGCGTTGGTGCAGGGCACCGAGATGAACACCACAGGGCTATTTGCCTGTC
O.barthii           CCGCCAACGTGGCGTCGGTGCAGGGCACCGAGATGAACACCACGGGGCTATTTGCCTGTC
O.rufipogon         CCGCCAACGTGGCGTCGGTGCAGGGCACCGAGATGAACACCACGGGGCTATTTGCCTGTC
O.nivara            CCGCCAACGTGGCGTCGGTGCAGGGCACCGAGATGAACACCACGGGGCTATGTGCCTGTC
O.longistaminata    CCGCCAACGTGGCGTCGGTGCAGGGCACCGAGATGAACACCACGGGGCTATTTGCCTGTC
                     ******* ****** ********************  ****  ******* ********

Zhonghua11          CAGGGTCATCCTAGGCTTAAGGCCACGACACTCAAGGACGTGGTGGGCGGCGTCACAGAG
Nipponbare          CAGGGTCATCCTAGGCTTAAGGCCACGACACTCAAGGACGTGGTAGGCGGCGTCACAGAG
9311                CAGGGTCATCCTAGGCTTAAGGCCACGACACTCAAGGACGTGGTCGGCGGCGTCACAGAG
MH63                CAGGGTCATCCTAGGCTTAAGGCCACGACACTCAAGGACGTGGTGGGCGGCGTCGCGGAG
R498                CAGGGTCATCCTAGGCTTAAGGCCACGACACTCAAGGACGTGGTGGGCGGCGTCGCGGAG
ZS97                CAGGGTCATCCTAGGCTTAAGGCCACGACACTCAAGGACGTGGTGGGCGGCGTCGCGGAG
O.meridionalis      CAGGGTCATCCCAGGCTCAAGGCCACGACACTCAAGGACGTGGTGGGCGGTGTTGCGGAG
O.glumaepatula      CAGGGTCATCCTAGGCTTAAGGCTACGACACTCAAGGACGTGGTGGGCGGCGTCGCGGAG
O.barthii           CAGGGTCATCCTAGGCTCAAGGCCACAACACTCAAGGACATGGTGGGCGGCGTCGCGGAG
O.rufipogon         CAGGGTCATCCTAGGCTTAAGGCCACGACACTCAAGGACGTGGTAGGCGGCGTCACAGAG
O.nivara            CAGGGTCATCCTAGGCTTAAGGCCACGACACTCAAGGACGTGGTGGGCGGCGTCGCGGAG
O.longistaminata    CAGGGTCATCCTAGGCTCAAGGCCACGACACTCAAGGACGTGGTGAGCGGCGTCGCGGAG
                    *********** ***** ***** ** ************ ****  **** **  * ***

Zhonghua11          GTGCTCCCAGCGAACAAGCTGGCCACCAAGGAGGACGCCGACAAGGTGGCGGCCACCGCT
Nipponbare          GTGCTCCCAGCGAACAAGCTGGCCACCAAGGAGGACGCCGACAAGGTGGCGGCCACCGCT
9311                GTGCTCCCAGCGAACAAGCTGGCCACCAAGGAGGACGCCGACAAGGTGGCGGCCACCGCT
MH63                GTGCTCCCAGCGAACAAGCTGGCCACCAAGGAGGACGCCGACAAGGTGGCGGCCACCGCT
R498                GTGCTCCCAGCGAACAAGCTGGCCACCAAGGAGGACGCCGACAAGGTGGCGGCCACCGCT
ZS97                GTGCTCCCAGCGAACAAGCTGGCCACCAAGGAGGACGCCGACAAGGTGGCGGCCACCGCT
O.meridionalis      GTGCTCCCAGCGAACAAGTTGGCCACCAAGGAGGACGCCGACAAGGTGGCGGCCACCGCT
O.glumaepatula      GTGCTCCCAGCGAACAAGCTGGCCACCAAGGAGGACGCCGACAAGGTGGCGGCCACCGCT
O.barthii           GTGCTCCCAGCGAACAAGCTGGCCACCAAGGAGGACGCCGACAAGGTGGCGGCCACCGCT
O.rufipogon         GTGCTCCCAGCGAACAAGCTGGCCACCAAGGAGGACGCCGACAAGGTGGCGGCCACCGCT
O.nivara            GTGCTCCAAGCGAACAAGCTGGCCACCAAGGAGGACGCCGACAAGGTGGCGGCCACCGCT
O.longistaminata    GTGCTCCCAGCGAACAAGCTGGCCACCAAGGAGGACGCCGACAAGGTGGCGGCCACCGCT
                    ******* ********** *****************************************

Zhonghua11          ATGCAGAAACGATGGGAGGCATGCCGGTGACGACAAGGAGTTAACACGATCCATTTAGTC
Nipponbare          ATGCAGAAACGATGGGAGGCATGCCGGTGACGACAAGGAGCTAACACGATCCATTTAGTC
9311                ATGCAGAAACGATGGGAGGCATGCCGGTGACGACAAGGAGCTAACACGATCCATTTAGTC
MH63                ATGTAG-AACGATGGGAGGCACGCCGGTGACGACAAGGAGCTAACACGATCCATTTAGTC
R498                ATGTAG-AACGATGGGAGGCACGCCGGTGACGACAAGGAGCTAACACGATCCATTTAGTC
ZS97                ATGCAG-AACGATGGGAGGCATGCCGGTGACGACAAGGAGCTAACACGATCCATTTAGTC
O.meridionalis      ATGCAG-AACGATGGGAGGCATGCCGGTGACGACGGGGAGCTAACACGATCCATTCAGTC
O.glumaepatula      ATGCAG-AACGATGGGAGGCACGCCGGTGACGACAAAGAGCTAACATGATCCATTTAGTC
O.barthii           ATGCAG-AATGATGGGAGGCACGCCGGTGACGACAAGGAGCTAACACGATCCATTTAGTC
O.rufipogon         ATGCAGAAACGATGGGAGGCATGCCGGTGACGACAAGGAGCTAACACGATCCATTTAGTC
O.nivara            ATGCAG-AACGATGGGAGGCACGCCGGTGACGACAAGGAGCTAACACGATCCATTTAGTC
O.longistaminata    ATGCAG-AACGATGGGAGGCACGCCGGTGACGACAAGGAGCTAACACGATACATTCAGTC
                    *** ** ** *********** ************   *** ***** *** **** ****

Zhonghua11          CCGATCCGAGTTTATCAGGAATTCAATCCTGCACCGTGCGGTTACG
Nipponbare          CCGATCCGAGTTTATCAGGAATTCAATCCTGCACCGTGCGGTTACG
9311                CCGATCCGAGTTTATCAGGAATTCAATCCTGCACCGTGCGGTTACG
MH63                CCGATCCGAGTTGATCAGGAATTCAATCCTGCACCGTGCGGTTACG
R498                CCGATCCGAGTTGATCAGGAATTCAATCCTGCACCGTGCGGTTACG
ZS97                CCGATCCGAGTTGATCAGGAATTCAATCCTGCACCGTGCGGTTACG
O.meridionalis      CCGATCTGAGTTGATCAGGAATTCAATCCTGCACCGTGTGGTTACG
O.glumaepatula      CCGATCTGAGTTGATCAGGAATTCAATCCTGCACCGTGCGGTTACG
O.barthii           CCGATCCAAGTTGATCAGGAATTCAATCCTGCACCGTGCGGTTACG
O.rufipogon         CCGATCCGAGTTTATCAGGAATTCAATCCTGCACCGTGCGGTTATG
O.nivara            CCGATCCGAGTTGATCAGGAATTCAATCCTGCACCTTGCGGTTACG
O.longistaminata    CCGATCCGAGTTGATCAGGAATTCAATCCTGCACCGTGCGGTTACG
                    ******  **** *********************** ** ***** *
```

Fig. S6 Multiple sequence alignment for the TTR region of *ESP*. The potential sites of cytosine methylation were highlighted. ▌ CG; ▌ CHG.

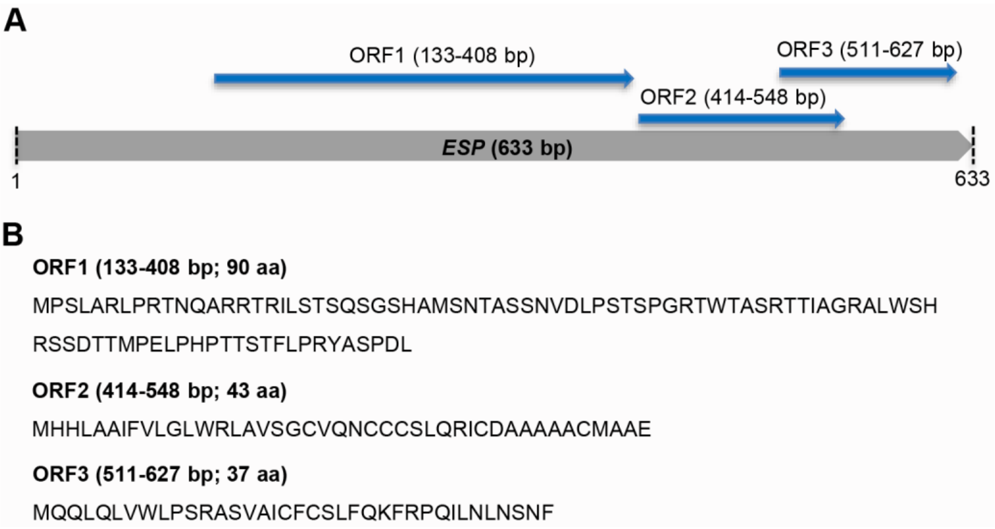

Fig. S7 Open reading frames (ORFs) prediction of *ESP* gene. **a** Overview of ORFs in the *ESP* sequence. **b** Amino acid (aa) sequences of the predicted polypeptides from three ORFs in (a).

Table S1 40 accessions of cultivated line.

Sample No.	Accession name	Origin
1	Mehr	Iran
2	Kalamkati	India
3	Jhona349	India
4	DZ78	Bangladesh
5	Binulawan	Philippines
6	Leung Pratew	Thailand
7	IR36	Brazil
8	Popot 165	Indonesia (E. Kalimantan)
9	Ai-Chiao-Hong	China
10	Guan-Yin-Tsan	China
11	Gie57	Vietnam
12	TD2	Thailand
13	JC91	India
14	Ta Hung Ku	China
15	Haginomae Mochi	Japan
16	Darmali	Nepal
17	Phudugey	Bhutan
18	Norin 20	Japan
19	Chodongji	South Korea
20	Mansaku	Japan
21	Nipponbare	Japan
22	Maintmolotsy	Madagascar
23	Jambu	Indonesia
24	Miriti	Bangladesh
25	AZUCENA	Philippines
26	NPE 844	Pakistan
27	Arias	Indonesia (West Java)
28	Gotak Gatik	Indonesia (C. Java)
29	Trembese	Indonesia (East Java)
30	Canella De Ferro	Brazil
31	Lemont	USA
32	Davao	Philippines
33	Kitrana508	Madagascar
34	Bico Branco	Brazil
35	JC101	India
36	JC111	India
37	Firooz	Iran
38	KUI SALI	Bangladesh
39	HAISHA CAMAN	Bangladesh
40	BADAL 89	Bangladesh

Table S2 Primers used in this study.

**Primers**	**Sequence (5′→ 3′)**	**Locus/Gene**	**Experiments**	**Marker's type**
RM583-F	AGATCCATCCCTGTGGAGAG	Chromosome 1	Map-based cloning	SSR
RM583-R	GCGAACTCGCGTTGTAATC			
RM23-F	CATTGGAGTGGAGGCTGG	Chromosome 1	Map-based cloning	SSR
RM23-R	GTCAGGCTTCTGCCATTCTC			
RM140-F	TGCCTCTTCCCTGGCTCCCCTG	Chromosome 1	Map-based cloning	SSR
RM140-R	GGCATGCCGAATGAAATGCATG			
RM493-F	TAGCTCCAACAGGATCGACC	Chromosome 1	Map-based cloning	SSR
RM493-R	GTACGTAAACGCGGAAGGTG			
RM513-F	TCTAGTGGCCTCAAAAAGGG	Chromosome 1	Map-based cloning	SSR
RM513-R	GCAACGAAATCATCCCTAGC			
RM466-F	TCCATCACCACATTCCCC	Chromosome 1	Map-based cloning	SSR
RM466-R	ACCCTTCTCTCGCTCTCTCC			
RM129-F	TCTCTCCGGAGCCAAGGCGAGG	Chromosome 1	Map-based cloning	SSR
RM129-R	CGAGCCACGACGCGATGTACCC			
RM446-F	ACAGCGAATACTCCAGACGG	Chromosome 1	Map-based cloning	SSR
RM446-R	TATCTCCCCCCAAATTCCTC			
RM9-F	GGTGCCATTGTCGTCCTC	Chromosome 1	Map-based cloning	SSR
RM9-R	ACGGCCCTCATCACCTTC			
AP003208-1F	TAGACAAAACGACCAAGGCCC	Chromosome 1	Map-based cloning	InDel
AP003208-1R	TCCAAGAGGTGCTAATGGGG			
AP003764-1F	CAGCCAAAGCATGGGCTACT	Chromosome 1	Map-based cloning	InDel
AP003764-1R	CAGGAGCCGGTAAAATCCCA			
AP003258-F	GGTCAGTTCAGCACTTATCTTGG	Chromosome 1	Map-based cloning	InDel
AP003259-R	AGCGGACAGAGACTTGGAGT			
AP003312-0F	CGTTCAGGAAAGTAGTGCAAGCC	Chromosome 1	Map-based cloning	InDel
AP003312-0R	TCCAATTGAGTATGTGTGCTCA			
AP003312-1F	TGATGATACATGGGTCACCAGT	Chromosome 1	Map-based cloning	InDel
AP003312-1R	GGTACTCGACTACTCTGCCC			
AP003204-2F	ACTCATTATGCCGCTCAGACA	Chromosome 1	Map-based cloning	InDel
AP003204-2R	AATCTCAGGGCATGACCACC			
OS01T0356900-01F	CACCGTTTTTCGAAGGATACTG	*Os01g0356900*	RT-PCR	/
OS01T0356900-01R	TCAATGTGGTTAAAGCATGAGC			
01G0356951-12F	ATCGCTATCTCGCCGAGTC	*ESP*	RT-PCR	/
01G0356951-462R	CAGCAAGTCTCCACAGTCCA			
OS01T0357100-02F	CGACGAACTTGTGAACCATTTT	*Os01g0357100*	RT-PCR	/
OS01T0357100-02R	CCAACACCGCAATTAACTGATA			
OS01T0357200-01F	TTTCGACGAGGAGGTATTCATC	*Os01g0357200*	RT-PCR	/
OS01T0357200-01R	CTCCCCTTGATCTTCAATTTGC			
Os04g03980F	ATGGTGCTCCAACACAGTGA	*YUCCA7*	RT-PCR	/
Os04g03980R	TGCCAATGTACTCCATCTCG			
ESP-BSF	CGTTGATAAGTTATTATTATAATTATCGGCG	TRR of *ESP*	DNA methylation analysis	/
ESP-BSR	CGTAACCGCACGATACAAAATTAAATTCCTA			
ESP-BSgF	CGTTGATAAGTCACTACTACAACCATCGGCG	TRR of *ESP*	DNA methylation analysis	/
ESP-BSgR	CGTAACCGCACGGTGCAGGATTGAATTCCTG			

File 1 Experimental procedures

Plant materials

A spontaneously occurring rice short panicle mutant *esp* was isolated from the *Oryza sativa* L. ssp. *Japonica* cultivar Zhonghua 11.

Map-based cloning

For map-based cloning of the *ESP* gene, 1129 individual plants showing a dwarf phenotype with defect in shoot apical meristem were selected from an F_2 population derived from a cross between the *esp* mutant and *indica* var Huajingxian74. Bulk segregant analysis (BSA) was first performed for preliminary genetic mapping (Michelmore et al., 1991). Simple sequence repeats (SSRs) were identified using SSRHunter software (Li and Wan, 2005). For fine mapping, insertion-deletion (InDel) markers were obtained according to the rice DNA polymorphic database between Nipponbare (*O. sativa* ssp. *japonica*) and 93-11 (*O. sativa* ssp. *indica*) (Shen et al., 2004). Genomic DNA was extracted from young leaves of each parent and F_2 individuals using a convenient method (Zhang et al., 2013). To find out the mutation site, we amplified the corresponding fragments from the *esp* mutant and wild-type plants, respectively. Primers used for the map-based cloning were listed in Supplementary Table 2.

Gene expression analysis

Total RNA was extracted from frozen samples using TRIzol reagent (Invitrogen) according to the manufacturer's instructions. After RNase-free DNase treatment, and first strand cDNA was generated using a Revert Aid First Strand cDNA Synthesis Kit (Thermo Scientific). Quantitative RT-PCR (qRT-PCR) was performed using a SYBR Premix Ex Taq™ RT-PCR kit (Takara) following the manufacturer's instruction. The primers for qRT-PCR are shown in Supplementary Table 2. The rice *25sRNA* gene was used as an internal control for all analysis. Three replicates were performed for each analysis.

Bisulfite sequencing

Genomic DNAs were isolated from young panicle of the heterozygous *Epi-sp* (+/-) mutant or shoot apical meristem of the homozygous *Epi-sp* (-/-) mutant at heading stage. Bisulfite treatment of genomic DNA was conducted according to the previous methods descripted by zhang et al (2015). The primers used for bisulfite sequencing are listed in Supplemental Table S2.

5-aza-dC treatment

Rice seeds were soaked in water at 30°C for 24 h. The seeds were then immersed in 20 mM Tris-HCl, pH 7.5, with or without 0.3 mM 5-aza-dC (A3656; Sigma) at 28°C for 72 h in the dark. After washing, seeds were planted in soil. Seven-day-old seedlings were sampled for further analysis.

Sequence alignment

The sequences of *ESP* and its transcriptional termination region (TTR) were downloaded from RAP-DB (https://rapdb.dna.affrc.go.jp/) and used to search the rice genome database including Ensemble Genomes (http://ensemblgenomes.org/info/genomes) and Rice Information GateWay (RIGW, http://rice.hzau.edu.cn/rice/). Multiple sequences alignments were performed using MUSCLE (https://www.ebi.ac.uk/Tools/msa/muscle/) with default parameters.

References

Michelmore RW, Paran I, Kesseli RV (1991) Identification of markers linked to disease-resistance genes by bulked segregant analysis: a rapid method to detect markers in specific genomic regions by using segregating populations. Proc Natl Acad Sci USA 88: 9828-9832

Shen Y, Jiang H, Jin J, Zhang Z, Xi B, He Y, Wang G, Wang C, Qian L, Li X, Yu Q, Liu H, Chen D, Gao J, Huang H, Shi T, Yang Z (2004) Development of genome-wide DNA polymorphism database for map-based cloning of rice genes. Plant physiol 135: 1198-1205

Zhang X, Hou P, Zhu H, Li G, Liu X, Xie X (2013) Knockout of the VPS22 component of the ESCRT-II complex in rice (*Oryza sativa* L.) causes chalky endosperm and early seedling lethality. Mol Biol Rep 40: 3475-3481

Zhang X, Sun J, Cao X, Song X (2015) Epigenetic mutation of *RAV6* affects leaf angle and seed size in rice. Plant Physiol 169: 2118-2128
